# Research on Transfer Optimization Model of County Transit Network

**DOI:** 10.3390/ijerph18094962

**Published:** 2021-05-07

**Authors:** Xu Sun, Kun Lin, Pengpeng Jiao, Zelin Deng, Wei He

**Affiliations:** 1 School of Civil and Transportation Engineering, Beijing University of Civil Engineering and Architecture, Beijing 100044, China; linkun@stu.bucea.edu.cn (K.L.); jiaopengpeng@bucea.edu.cn (P.J.); dengzelino@163.com (Z.D.); hewei@bucea.edu.cn (W.H.); 2 Institute of Transportation Engineering, Tsinghua University, Beijing 100084, China

**Keywords:** transit network, layout mode, transfer optimization, synchronization reach, operation management, smart mobility

## Abstract

County transit is an important mode that connects the county center with the surrounding countryside. This paper addresses the problem of unreasonable transit network planning, inconvenient operational optimizations, and protections in the country transit network system to build the transfer optimization model of the county transit network. The model that maximizes the synchronization reach operates in the “end-point connection”, which is the most suitable layout mode by analyzing the characteristics of county transit passenger flow and for comparing different layout modes. Typical county-level cities in three urban agglomerations in China were chosen as cases to validate the effectiveness and practicability of the proposed model. The case results are compared and analyzed in terms of the network density, departure interval, county population, and economic development level, which give theoretical support for decision-making in the planning, construction, and operation management of public transportation in China’s counties.

## 1. Introduction

A county is a unique administrative division in China with a predominantly urban population and a relatively concentrated mobile population, as well as being a distribution point for nearby villages with an average level of urbanization. The economy of a county lags behind that of a city, and its population density is greater than that of the countryside. The county has obvious urban structure layout and resident’s distribution characteristics, resulting in a unique urban road network structure, which is widely distributed in China and has high research value. A county is the geographical scope of the county administrative division, including the central city, the surrounding towns, and administrative villages. There are a large number of towns and villages in China’s counties, most of which have poor traffic connectivity and are inconvenient to the central city. With the rapid economic development in counties, it is urgent to equip the transit network with a high coverage rate and density to facilitate the county residents’ trip. Now, most central cities of the counties have a comprehensive public transportation system, while the surrounding towns and villages are relatively inferior. Therefore, the paper argues that national strategies should be made to strengthen urban and rural public transport, to promote the transit transfers between the central city and villages by the transit extension from urban to rural areas.

The county consists of the cities, towns, and villages within its boundaries, where the overall development has not reached the level of urban development. There is a lack of public transportation infrastructure and an economic and travel dissonance between the city and the surrounding areas. The evidence referred to above indicate that the county transit system needs further research, including the integration of central city and village bus routes, the optimization of its layout mode as well as the transfer model. The development of efficient transfer optimization and operation management will enable the rational connection of bus routes among the central city and villages, formulating a fast, orderly, and comfortable county transit network. This will improve the convenience of connecting different bus routes within the county and maximize the use of transit resources so that a bus system, fitting for the county’s development, can be established to solve the divorce of cities and rural areas.

The development of urban transport is different between western countries and China due to the smaller country transit systems in western countries, so researchers have emphasized the development of transit optimization models and algorithms [1,2,3,4,5,6]. Initial research on bus network planning focused on single routes or small-scale road networks with simple models that lacked applications [7,8]. However, as the research progressed, the study of network planning rose from the line level to the road network level [9,10,11,12]. Bus network planning is a NP-hard (non-deterministic polynomial, NP) problem, which is extremely complex and difficult to solve using traditional algorithms, while the intelligent optimization algorithm is often used to solve it due to its iterative optimization properties [13,14]. It is necessary to consider bus interchange in the process of bus network planning, but the existing studies overly focus on bus interchange, especially on reducing the number of interchanges and shortening the interchange time through various algorithms, without paying sufficient attention to interruptions and continuity of interchanges [15]. In order to improve the operational efficiency of the bus system, many scholars have also optimized bus schedules with the help of interchange windows [16], dynamic planning [17], and vehicle networking [18], modeled for the passenger waiting time [19], bus operation cost [20], and maximum passenger flow [21], and solved the problem by genetic algorithm [22], particle swarm optimization [23], hybrid differential evolution and neural networks [24], which have made progress in bus departure interval design and greatly optimized the performance of bus scheduling systems, but rarely considered the impact of passenger interchange in optimizing schedules.

As can be seen, the bus route network planning model and algorithm have been analyzed in depth, which not only achieves more theoretical results but can also be applied to some cities, improving the reliability of bus scheduling systems and the efficiency of urban bus operations, and shortening the waiting and transfer time for residents. However, most studies are focused on urban public transportation, with the optimization of bus route planning methods, bus schedules and vehicle dispatching, and lack of research on county public transportation [25]. Compared with urban areas, the urban-rural areas are widely spread with lower and unevenly distributed passenger demand, different passenger flow distribution situation and scale, and obvious differences in travel purposes, so it is improper to apply the results of urban bus research directly to county buses. In addition, the regional specificity of the county makes the route layout pattern, bus stop setting method, and transfer characteristics of the county bus different from those of the urban bus, so the study of bus network planning must be conducted based on the special characteristics of county bus.

In this article, the layout patterns, transfer optimization and security measures of county transit network systems in China have been studied. By analyzing passenger flow characteristics and comparing the layout modes, the most suitable layout mode is selected for the county transit network to determine specific optimization goals of this network and establish a transfer optimization model. Compared with the existing model, the model is in line with the form of line network layout of the county network, and improves the efficiency of the residents’ travel interchange effectively, which has put forward new ideas for the study of county bus interchange. Additionally, the application of intelligent algorithms improves the calculation accuracy of the model. Besides, suggestions were given for the operation and management of county public transportation based on this case, providing theoretical support for the planning and design of transit in China’s counties. This paper develops an efficient line network interchange optimization and operation management programs to improve the bus routes in towns and villages in the county, so that the bus routes can be reasonably connected to form a fast, orderly, and comfortable county bus network, and ultimately improving the traffic situation in the county.

The remainder of this article is organized as follows. Section 2 selects “end-point connection” in county transit layout and provides the transfer optimization model formulation of a mixed integer programming problem. Section 3 proposes an intelligent optimization algorithm that solves this problem. Case studies based on real-world road networks are introduced and solved to prove the application of the proposed algorithm in Section 4. Finally, conclusions and extensions are presented in Section 5.

## 2. The Transfer Optimization Model of County Transit Network

### 2.1. The Mode Selection of County Transit Layout

There are mainly radial, square and radio-ring networks in the existing urban public transportation network layouts, which are divided according to the urban layout structure. However, the county transportation network layout is different from the conventional urban network because of the differences in economics, traffic size, and travel demand between the city center and nearby towns. Therefore, the layout modes of county transit are mainly divided into three groups: “radius linear”, “pulling-through”, and “end-point connection”.

The radius linear layout mode connects the city center and towns in the county directly, in which the pressure is concentrated in the central city and causes congestion. The Pulling-through layout mode is crossed from the center of the city to towns and villages with some stops, which can maximize the direct transportation of county passengers and reduce the total number of transfers. However, it will take up more road resources in the central area, and some lines have a high repetition factor, resulting in wasted road resources. End-point connection layout mode can connect the town and the outside of the city, and country passengers indirectly enter the city center by transferring to city buses. The advantage is that country buses do not pass directly through the city center, there is little impact on urban traffic pressure; and the total length of the line is short to facilitate flexible operation and maintenance. The disadvantage is that the directness is worse than the radial line and through layout, forcing most passengers to transfer before they reach their destination the main characteristics of which are illustrated in Table 1.

As shown in Table 1, it can be seen that no modes can make all indicators, including traffic pressure, directness, service level, and organization difficulty to achieve the optimal stage at the same time. They all have shortcomings: the “radius linear” concentrates on greater pressure on the central city, the “end-point connection” has lower accessibility, and the long route of “pulling-through” is likely to increase route repeatability. There are obvious differences in traffic congestion and the residents’ travel demand between the central city and villages. For example, the traffic pressure on the central city is higher than in other areas, and the demand for public transport for urban residents is mainly for short distances, while people living in villages prefer long-distance travel that connects villages and the central city. Therefore, village lines should not pass the city center to avoid additional traffic pressure on the central city when planning the network layout.

**Figure 1:**
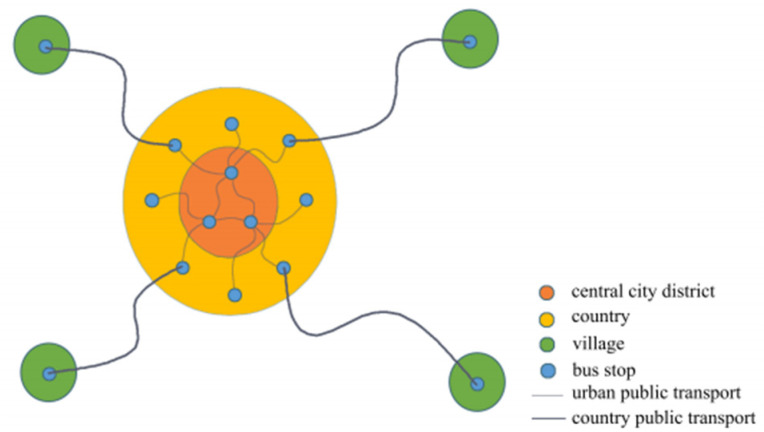
End-point connection.

As shown in Figure 1, the obvious characteristics of end-point connection lines are not directly passing the central city with less pressure on the urban traffic, and lines that are short with flexible operation. In contrast, radius linear and pulling-through lines both run through the central city, which will increase the traffic pressure there. Moreover, the pulling-through lines are too long to maintain a high service level, and it increases operation management cost.

Therefore, the end-point connection is the most suitable layout mode for the county transit, which has important implications for developing the optimization model. Considering the relatively poor directivity of the end-point connection, the departure frequencies and departure times of bus lines can be adjusted during the modeling process to mitigate this problem to some extent.

### 2.2. Notations

The notations used in the transfer optimization model of county transit network are presented in Table 2.

### 2.3. Model Formulation

The advantages of the end-point connection are no additional traffic pressure on the county central city and less difficulty in operation management, which reduces pressure on the construction and management. While this layout mode provides less direct access, the transfer optimization model should be established to reduce the number of passenger transfers and transfer waiting times in the country. The optimization goal is to determine buses in different levels of arrival at the transfer station at the same time and to shorten the time difference between buses that cannot arrive synchronously, so as to get the maximum number of simultaneous arrivals within a certain period.

The objective of the model is formulated as follows:

(1) $$\begin{array}{l} max\sum_{z_{1}=1}^{3}\sum_{l=1}^{M_{z_{1}}}\sum_{i=1}^{F_{z_{1}l}}\sum_{z_{2}=z_{1}+1}^{3}\sum_{m=1}^{M_{z_{2}}}\sum_{j=1}^{F_{z_{2m}}}\sum_{x\in A_{z_{1}z_{2m}}}S_{z_{1}liz_{2}mjx}\\ \;\;\;\;\;\;s.t. \end{array}\;H_{minl}\leq H_{maxl},$$

(2) $$\left(F_{zl}-1\right)\cdot H_{minl}\leq T\leq F_{zl}\cdot H_{maxl},$$

(3) $$X_{1zl}\leq H_{maxzl},\;1\leq l\leq M_{z},$$

(4) $$X_{F_{zl}}\leq T,1\leq l\leq M_{z},$$

(5) $$\;\;\;\;\;\;\;\;\;\;\;H_{minzl}\leq X_{\left(i-1\right)zl}-X_{izl}\leq H_{maxzl},1\leq l\leq M_{z},1\leq i\leq F_{zl}-1.$$

The constraints of the model are formulated from Equations (1) to (5). Constraint (1) ensures that the first departure interval would not exceed the maximum allowable headway. Constraint (2) assures that the first departure of each bus route l must be in the $\left[0,H_{maxl}\right]$ and the frequency, an integer, is determined by the departure interval. Constraint (3) indicates that the first departure is within the optimization interval. Constraint (4) guarantees that the last departure cannot exceed the maximum allowable headway from the start of the optimization interval. Constraint (5) limits the departure interval of two adjacent buses. The constraint is a nonlinear constraint and cannot be solved by linear programming. In addition, this problem is a non-linear programming problem with the objective of maximizing the number of simultaneous interchanges, which is a complex problem and takes a long time to solve. Therefore, in order to improve the solution efficiency, the heuristic algorithm is chosen.

## 3. Intelligent Optimization Algorithm

The purpose of the transit network optimization model of the county transit network is to maximize the number of simultaneous arrivals in a certain period. Earlier studies of bus networks are based on simple road network design, with few considerations and simple models, which are mainly solved by traditional optimal methods. The small scale of the model is a mixed integer linear programming problem solved with optimization software such as Lingo, while the large scale is a mixed-integer programming problem requiring hours and even days to solve. Intelligent algorithms, due to their iterative optimization nature, usually find the global optimal solution in the short term by simulating natural processes. In order to improve efficiency, the transfer optimization model is solved by the intelligent optimization algorithm, which sets up departure time based on the purposeful selection of nodes in the network. Departure times are added if the line does not satisfy the departure frequency.

The intelligent optimization algorithm can be summarized with the following steps:

**Step 1:** Node selection.

At the first selection, the maximum traveling time of all nodes is arranged in descending order. The smallest value node is selected, then the next node in a descending order is chosen.

**Step 2:** Time difference and departure time determinations.

Two bus lines, l and m , correspond to intervals $H_{minl},\;H_{maxl},\;H_{minm},\;H_{maxm}$, and calculate $H_{mind}=max\left(H_{minl},H_{minm}\right)$, $H_{maxd}=min\left(H_{maxl},H_{maxm}\right)$.

(i) If $H_{mind}>H_{maxd}$, two lines cannot form a stable difference and can only set the first traveling time. The shorter traveling time is set to 0 and the other lines’ departure time is set to meet at the node.
(ii) If $H_{mind}\leq H_{maxd}$, $d=H_{mind}$ is chosen as the stable difference of two buses at the same line.$\;d=H_{mind}$ is added to the first departure time to ensure each bus meets at the same node, which maximizes the number of encounters. When accumulation reaches the maximum number, departure times and numbers of simultaneous arrival are recorded.

**Step 3:** Departure time addition

(i) Add a line departure time and set the first departure time to ensure at least one simultaneous arrival. Then, select the appropriate time interval between the minimum and maximum time interval to form at least one simultaneous arrival. If this is impossible, select the minimum time interval to do the accumulation.
(ii) Add both lines’ departure time, then add the synchronization time as in step 2.

**Step 4:** Departure frequency determination

If it satisfies departure frequency, this algorithm stops; otherwise, fill in asynchronous time. There are three conditions:

(i) The bus line schedule has large time gaps. Then, appropriate time points are added between gaps.
(ii) The bus line schedule does not have large time gaps. Then, fill in the minimum departure interval in the end, which can satisfy departure frequency or reach the edge of the optimization interval.
(iii) When many unset departure times still exist at the edge of the optimization interval, the last departure time will be removed, and the departure interval will be shortened to reschedule the bus timetable until the schedule is complete.

**Figure 2:**
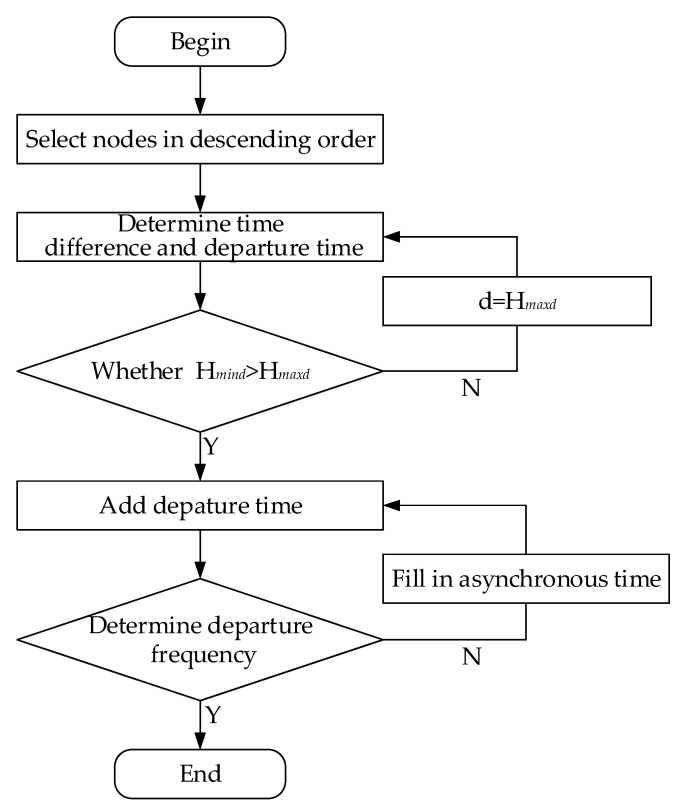
The flowchart of the intelligent optimization algorithm.

The flowchart of the intelligent optimization algorithm is illustrated in Figure 2.

## 4. Case Study

### 4.1. Example Network Configuration

**Figure 3:**
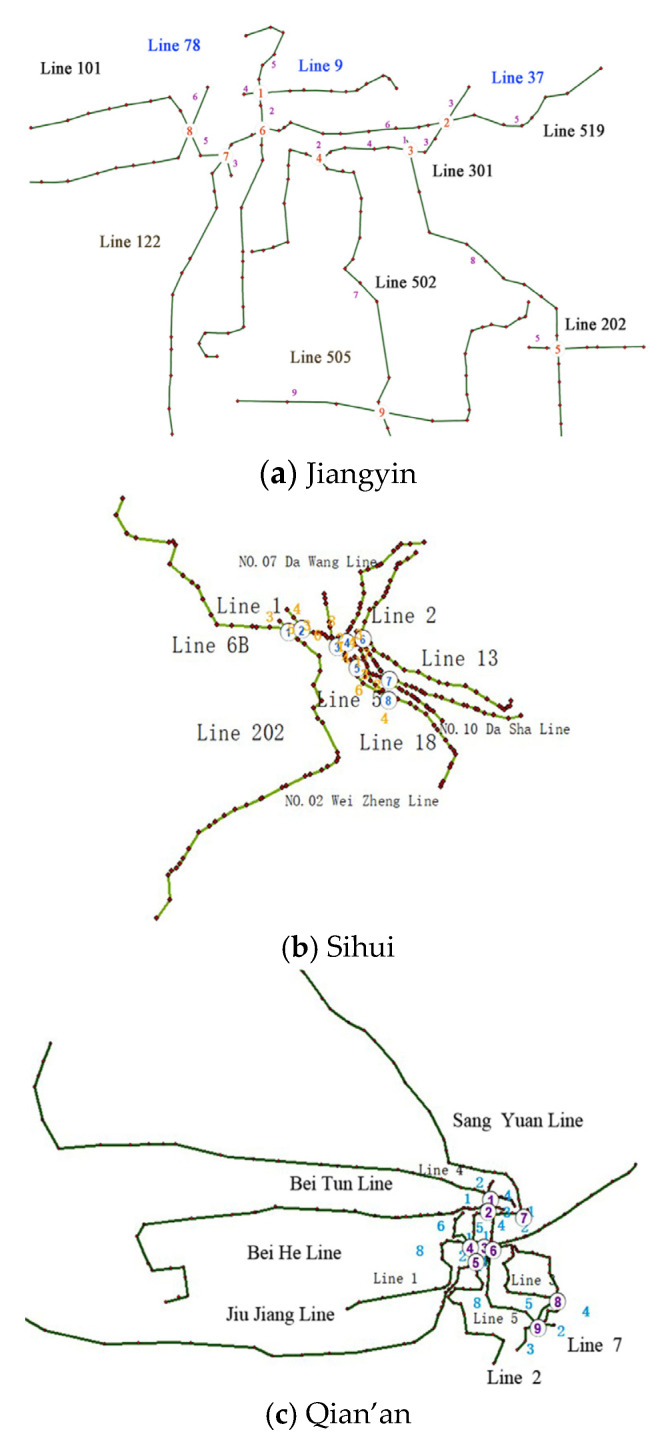
Transit networks.

Three typical counties, Jiangyin, Sihui, and Qian’an, from the Chinese City Clusters “Yangtze River Delta”, “Pearl River Delta”, and “Beijing-Tianjin-Hebei” are selected to verify the applicability and feasibility of the proposed model and algorithm. The basic characteristics and transit network are shown in Table 3 and Figure 3, respectively.

### 4.2. Case Calculation

**Figure 4:**
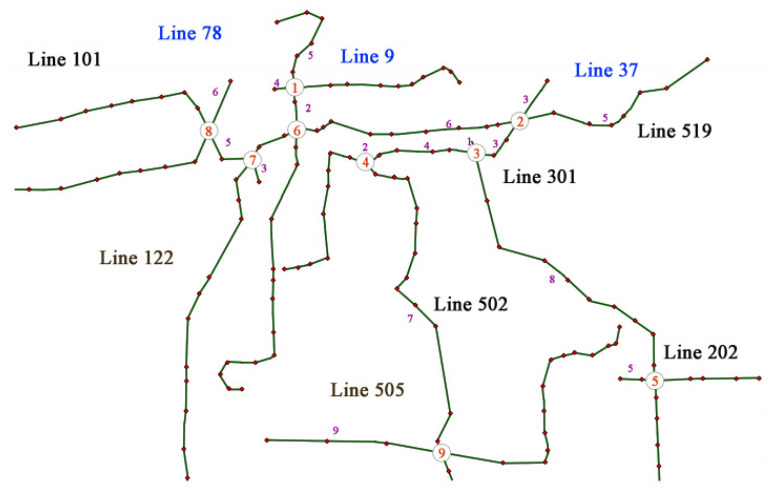
The basic network for Jiangyin.

Excluding some duplicate and low utilization routes, 10 main bus routes in Jiangyin county were selected for analysis, which included nine interchange stations, as shown in Figure 4. The number on the arcs is traveling time (in minutes) and the optimization intervals are discrete at [0, 60 min].

The departure interval and departure frequency for Jiangyin are presented in Table 4.

**Step 1:** Node selection:

Determine the filter order of the nodes. The maximum arrival times of nine nodes are arranged from small to large: 5, 5, 6, 10, 5, 11, 12, 6, 9 to get the corresponding node order: ①②⑤③⑧⑨④⑥⑦.

**Step 2:** Time difference and departure time determinations:

- Node ① takes the time difference d = 6. Departure times of Line 78 are 0, 6, 12, 18, 24, 30, 36, and Line 5 are 1, 7, 13, 19, 25, 31, 37, 43, 49. Synchronization time S = 5.
- Node ② takes the time difference d = 10. Departure times of Line 519 are 0, 10, 20, 30, 40, and Line 37 are 2, 12, 22, 32, 42, 52. Synchronization time S = 7 + 5 = 12.
- Node ⑤ takes the time difference d = 12. Departure time of Line 301 are 0, 12, 24, 36, 48, and Line 37 are 4, 16, 28, 40. Synchronization time S = 12 + 4 = 16.
- Node ③ is the node of line 37 and line 301, and the departure time of both lines has been set without simultaneous arrival. The number of synchronization S remains 16.
- Node ⑧ takes the time difference d = 12. Departure times of Line 101 are 0, 12, 24, 36, 48, and Line 122 are 2, 14, 26, 38. Synchronization time S = 16 + 4 = 20.
- Node ⑨ takes the time difference d = 10. Departure times of Line 505 are 0, 10, 20, 30, 40, and Line 502 are0, 10, 20, 30, 40, 50. synchronization time S = 20 + 5 = 25.
- Node ④ is the node of line 37 and line 502, and the departure time of both lines has been set with 5 simultaneous arrives. Synchronization time S = 25 + 5 = 31.
- Node ⑥ is the node of line 78 and line 519, and the departure time of both lines has been set with 1 simultaneous arrive. Synchronization time S = 30 + 1 = 31.
- Node ⑦ is the node of line 101 and line 519, and the departure time of both lines has been set without simultaneous arrival. The number of synchronization S remains 31.

**Step 3:** Departure time addition:

Set additional departure time of Line 37 and Line 301. When the departure time of line 37 is 52, it adds a departure time of 57 for Line 301 to create 1 simultaneous Synchronization time S = 31 + 1 = 32.

**Step 4:** Departure frequency determination:

The 10 lines of the Jiangyin bus all meet the requirements, so the departure timetable has been set up.

Using the four above steps of the intelligent optimization algorithm, the departure timetable of the transit network in Jiangyin is presented in Table 5.

The synchronization timetable is listed in Table 6, with a detailed calculation of a 10 line timetable for Jiangyin.

**Figure 5:**
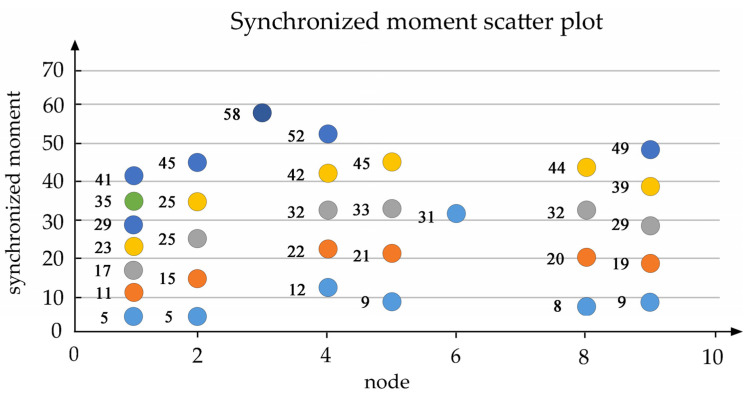
Jiangyin synchronized moment scatter plot.

The scatter plot of Figure 5 reveals that there has been a visual relationship between the synchronized time and node in Jiangyin.

Based on the same calculation process, departure timetables of the transit network in Sihui and Qian’an are shown in Table 7 and Table 8, respectively.

### 4.3. Results and Discussions

According to the calculation results of the 10 lines selected from the three cases of Jiangyin, Sihui and Qian’an, the number of synchronizations are 32, 20, 25, respectively, in the interval of [0, 60 min]. Therefore, the transfer efficiency of bus lines can be obtained: Jiangyin > Qian’an > Sihui.

The reasons are analyzed as follows:

(1) Transit network density

**Figure 6:**
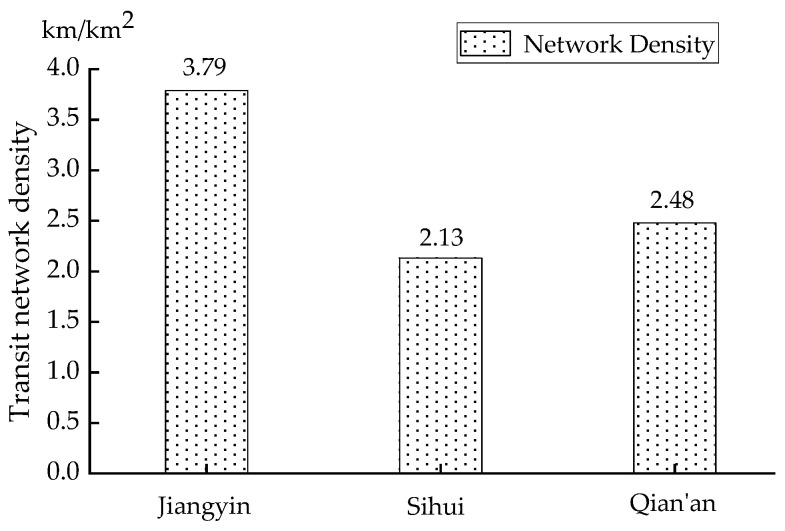
Network density and the number of synchronizations.

There should be a positive correlation between the number of synchronization and the county transit network density because the transit network density reflects the proximity of county residents to public transportation. The greater density of the transit network, the more convenient it is for county residents to use public transportation. The number of synchronization times in the three cities show a positive trend concerning the density of the transit network in Figure 6. It can be seen from this figure that the network density in Jiangyin is 3.79 km/km^2^, which is between 3 and 4 km/km^2^, meeting the criteria for the central city; the network densities in Sihui and Qian’an are 2.13 km/km^2^ and 2.48 km/km^2^, respectively, which are between 2 and 2.5 km/km^2^, meeting the standard of the urban edge. Therefore, cities with relatively low network density, such as Sihui and Qian’an, should strengthen the line coverage of transit network and especially improve the connectivity lines to ensure that all administrative villages are indirectly connected to public transport in the central city and promote smooth connectivity. Cities with high network density such as Jiangyin should maintain the original network and do resident trip surveys regularly to build new bus lines.

(2) Departure interval

**Figure 7:**
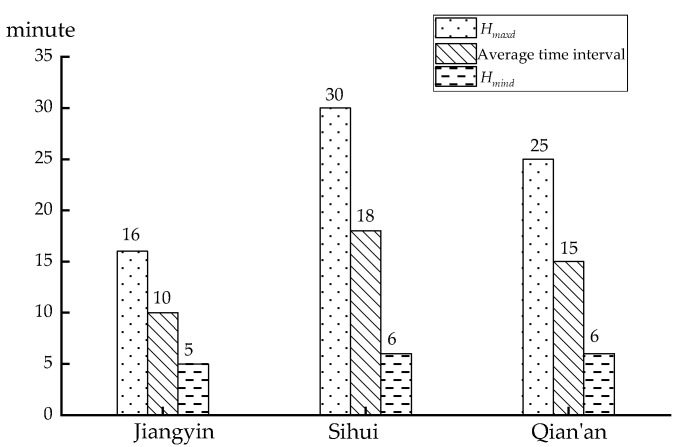
Departure interval of cases.

The number of synchronizations is negatively associated with the departure interval and a probable explanation is that the departure interval can reflect the efficiency of the system during the operation of the transit system. If the departure interval is short, passengers’ waiting time at the transfer stop is relatively short and the operating efficiency of this system is high. The maximum departure interval, the average departure interval, and the minimum departure interval of the three cities are compared in Figure 7, in which the number of synchronizations show a negative correlation with the interval time. Jiangyin has the smallest bus departure interval and the highest departure frequency, which indicates a higher the transfer efficiency, followed by Qian’an and then Sihui. Since departure interval changes will directly affect the number of synchronizations, the traveling time and departure interval should be collected and given real-time feedback to a bus dispatching center during operation, so that when the bus operating status changes, the bus traveling time can be obtained timely, which is useful for setting transfer time.

(3) Population size of counties:

The synchronization is positively associated with the travel residents. However, due to the lack of tourism personnel information collection agencies, the population size of the three counties is compared. It can be seen that the population size affects the density of bus routes and thus indirectly the efficiency of transfers.

**Figure 8:**
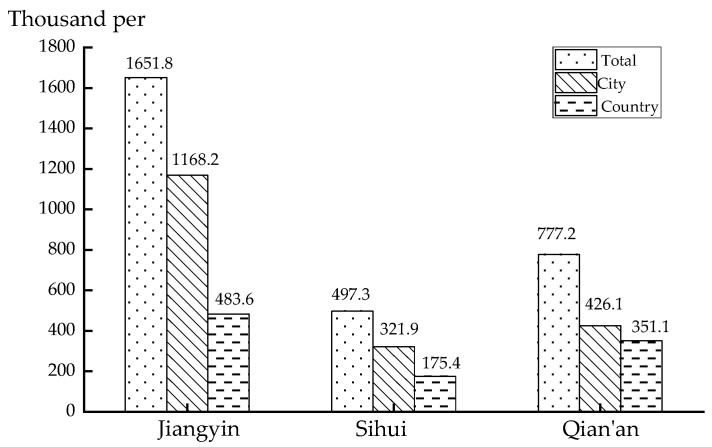
Population size of countries.

The total population, city population, and village population of the three cases are compared separately, as shown in Figure 8. There is a large difference in the total population between the three cases and Jiangyin is much higher than the other two cities, leading to higher network density and the highest transfer efficiency. Furthermore, there is a large population distribution in Jiangyin villages, which also improves the development of transit, so the overall synchronous transfer in Jiangyin is the most effective.

For counties that have a large proportion of the village population and good county economic, the government has successfully implemented the bus priority strategy to promote the village residents to choose public transportation to travel, and therefore these types of county transit systems operate better. On the other hand, the government failed to take bus priority strategy seriously in counties that have a small percentage of the village population and less economic development. Due to the lack of buses and low departure density, village residents reduce the original transit travel. It is difficult for the bus company to afford the operation cost of this system. Therefore, it is necessary to strengthen the public transport operation in counties with a small proportion of the village population and poor economic development.

**Figure 9:**
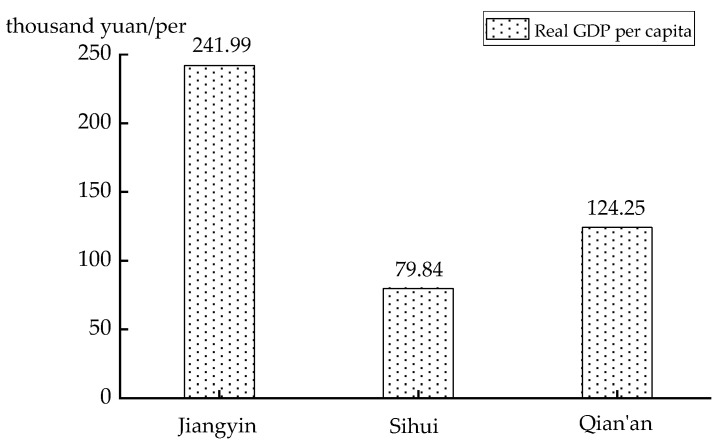
Per capita GDP of counties.

(4) Economic development level:

A possible explanation is that the number of synchronizations is a result of the economic development level. Economic development level indirectly affects the government’s investment in bus companies, which means that more investors are implemented in economically developed counties. Compared with the GDP, the most economically developed county is Jiangyin, with 400.112 billion yuan, GDP per capita 241,000 yuan; the second is Qian’an, with 96.54 billion yuan, GDP per capita 79,000 yuan; and the last is Sihui, with 39.705 billion yuan, GDP per capita 124,000 yuan. The economic disparity is also reflected in the transfer efficiency. From Figure 9 and the above study, it can be seen that Jiangyin has the highest economic level, the most mature urban public transportation, and the highest number of simultaneous interchanges; Qian’an, with only half the per capita GDP of Jiangyin, also has a significantly lower number of simultaneous transfers; Sihui, which has the lowest per capita economy, has only 60% of the number of simultaneous transfers of Jiangyin. Therefore, there is an important relationship between the development of urban economy and the number of simultaneous transfers in urban transportation.

Therefore, the government needs to invest more in the county transit system to implement line network expansion and facilities improvement. This is the most essential funding for improving the county’s transit system. It is also important to establish a transit subsidy mechanism for China’s counties, in which financial and non-financial subsidies complement each other, to ensure maximum efficiency. A series of subsides will be taken, such as direct subsidy funding, signal priority access, bus lane paving, and waiver of taxes (including vehicle purchase tax, fuel tax, and vehicle operation fee). Measures such as improving the mechanism for measuring subsidies, establishing a verification system for financial subsidies, and implementing the public transit priority policy will make the county transit subsidies system more reasonable and complete.

As shown in the above analysis, Jiangyin has the best development among the three counties as it has the largest population as well as public resources, more people travelling, and good interchange conditions, followed by Qian’an and Sihui. A larger population results in higher travel demand, which contributes to the infrastructure development and higher transit network density; while convenient interchange conditions increase to the probability of using public transportation, promoting the development of operating companies and regional economy. In underdeveloped regions, the lack of infrastructure necessary for travel makes it difficult to develop the economy, which leads to inefficient use of social resources. Therefore, in poor condition regions, the first step should be to improve interchange conditions in order to attract residents to use public transportation, thereby improving the connectivity and travel efficiency of the entire region.

## 5. Conclusions

The problems of the current country transit network system considered are unreasonable transit network planning, inconvenient operational optimization, and protections. This paper focuses on these problems and makes further research on the layout mode, transfer optimization, and security measures. By analyzing the characteristics of county transit passenger flow and comparing different layout modes, the most suitable layout mode for the transit network, ”end-point connection”, was proposed to be a modeling basis. In order to solve the poor directness of this layout, the transit transfer optimization model of county transit network is proposed, aiming to synchronize transfers in different lines and shorten the time difference between buses that cannot be reached synchronously. Then, the model was solved with the intelligent optimization algorithm, and applied to real networks of typical county-level cities in three urban agglomerations in China. The application results showed that the number of synchronizations from big to small were Jiangyin, Qian’an, and Sihui. The network density, county population, and economic development level were positively associated with the number of synchronizations, while the number of synchronizations had a negative relation to the departure interval. Analyzing this relationship, safeguard measures were put forward, which provided theoretical support and basis for the decision-making in planning, constructions, and operation management of public transportation in Chinese counties.

According to the layout structure and characteristics of the county, this paper proposes an “endpoint articulation” network layout model, which has been verified by actual engineering. The model is consistent with the characteristics of the county network, and better describes the connection between the urban center and the surrounding townships in the county area, which can improve the travel condition of the residents nearby towns, reduce the waiting time for transfers, shorten the travel time, and provide convenient services for travel. The problem is the complex simultaneous transfer problem between different level bus lines with calculations, so a heuristic algorithm is chosen to solve it to improve the efficiency of the model solution. According to the calculation results, four indexes were selected to analyze the effectiveness of the transfers, which provides an important reference for the counties to improve the layout of public transportation network.

There are still some limitations in this current research. One source of weakness in this study is considering only one central city in the county in the comparative analysis of the layout mode, which may fail to cover all county transit development modes in China. Further research should be extended to multiple central cities in the county when considering the layout mode. Another is that the bus traveling times on the arcs are indicated by the exact number, ignoring the influence of speed, road conditions, or other factors in actual travel. The calculated results can reduce the time difference of the transfer and may fail to develop complete synchronization arrival, and the probability distribution characteristics of the bus traveling time can be further considered in the future. Finally, in comparison with the traditional method, this paper uses intelligent algorithm to solve the model, which has obvious advantages in calculation speed and accuracy, and can greatly improve the transfer efficiency of residents’ travel and shorten the travel time and waiting time, but the research on the stability of calculation results needs to be strengthened. The existing results mainly focus on bus operation time, and research on the continuity of different travel mode interchanges can be conducted in the next step according to the characteristics of the county bus network.

## Figures and Tables

**Table 1:** Characteristic analysis of layout patterns.

Transit Mode	Network Layout	Traffic Pressure	Directness	Service Level	Organizational Difficulty
Radius linear	connects city and city outlying	increases pressure on the central city	high	high	low
Pulling-through	a connection between towns	increases pressure on the central city and route repetition	high	long routes; provides low services	high, increase operating cost
End-point connection	connects villages and city outlying, then transfers into the city	no press on the central city	low	high	low

**Table 2:** Notation definitions.

Symbol	Definition
$G=\{A,\bar{N}\}$	Set of the country transit network;
A	Set of arc meaning the traveling path of the bus line;
$\bar{N}$	Set of transfer nodes;
N	Number of transfer nodes;
T	Set of optimization time in the interval [0,T];
z	Bus line Level in country, city bus z = 1; town bus z = 2; village bus z = 3.
M	Number of bus line;
$M_{z}$	Number of bus line-level z;
$H_{minl},H_{maxl}$	minimum and maximum of the departure interval of bus line l and the departure time must in $\left[H_{minl},H_{maxl}\right]$;
$F_{zl}$	Number of departures for line l during the interval [0,T];
$T_{zlx}$	Traveling time from starting point in line l of level $\;z_{1}$ to node x. Not passing node x is represented by $T_{zlx}=-1$;
$X_{izl}$	The departure time of the i th bus in line l of level z;
$A_{z_{1}lz_{2}m}$	$A_{z_{1}lz_{2}m}=\left\{x:1\leq x\leq N,T_{z_{1}lx}\geq 0,T_{z_{2}mx}\geq 0\right\}$, if line l of level $\;z_{1}$ meets line m of level $\;z_{2}$ at node x, the variable is 1; otherwise, it yields the value 0.
$S_{z_{1}liz_{2}mjx}$	0–1 variable, when the i th bus in line l of level $\;z_{1}$ meets the j th bus in line m of level $\;z_{2}$ at node x, it yields the value 1; otherwise, the variable is 0; when $X_{iz_{1}l}+T_{z_{1}li}=X_{jz_{2}m}+T_{z_{2}mj}$, $S_{z_{1}liz_{2}mjx}=1$, otherwise, it yields the value 0.

**Table 3:** Basic features of cases.

Country	City Cluster	Contain Towns	Population (Million)	Line Classification and Number
Jiangyin	Yangtze River Delta	9	126.41	City Lines 8 Town Lines 25 Village Lines 72
Sihui	Pearl River Delta	10	49.73	City Lines 22 Town Lines 11
Qian’an	Beijing-Tianjin-Hebei	17	77.7	City Lines 17 Town Lines 20

**Table 4:** Departure interval and departure frequency for Jiangyin.

Line Level	Line Number	Setting Parameters		
		$H_{mind}$	$H_{maxd}$	Departure Frequency
City Lines	9	5	8	8
	37	7	10	6
	78	6	8	7
Town Lines	101	10	12	5
	202	12	16	4
	301	8	12	6
	502	6	10	6
	519	10	12	5
Village Lines	122	12	15	4
	505	10	13	5

**Table 5:** Departure timetable for Jiangyin.

Line Level	Line Number	Departure Time							
City Lines	9	1	7	13	19	25	31	37	43
	37	2	12	22	32	42	52		
	78	0	6	12	18	24	30	36	
Country Lines	101	0	12	24	36	48			
	202	4	16	28	40				
	301	0	12	24	36	48	57		
	502	0	10	20	30	40	50		
	519	0	10	20	30	40			
Village Lines	122	2	14	26	38				
	505	0	10	20	30	40			

**Table 6:** Synchronized timetable for Jiangyin.

Synchronized Time (Node)			
4 (①,②)	8 (⑧)	9 (⑤,⑨)	11 (①)
12 (④)	15 (②)	17 (①)	19 (⑨)
20 (⑧)	21 (⑤)	22 (④)	23 (①)
25 (②)	29 (①,⑨)	31 (⑥)	32 (④,⑧)
33 (⑤)	35 (①,②)	39 (⑨)	41 (①)
42 (④)	44 (⑧)	45 (②,⑤)	49 (⑨)
52 (④)	58 (③)		

**Table 7:** Departure timetable for Sihui.

Line Level	Line Number	Departure Time						
City Lines	1	0	10	20	30	40	50	60
	2	0	12	24	32	39	46	
	5	2	12	22	32			
	6B	1	11	21	31	41		
	13	3	19	35				
	18	3	9	15	21	27	33	
	202	6	20	34	48			
Country Lines	NO.02Weizheng line	0	13					
	NO.07Dawang line	1	13	25				
	NO.10Dasha line	15	30	45	60			

**Table 8:** Departure Schedule for Qian’an.

Line Level	Line Number	Departure Time							
City Lines	1	8	13	20	27	34	41	48	55
	2	1	10	19	28	37	46		
	3	0	9	18	27				
	4	0	14	28	42	56			
	5	2	17	32					
	7	4	13	22	31	40	49		
Country Lines	Binhe line	0	14	28					
	Sangyuan line	13	38						
	Beitun line	12	26	40					
	Jiujiang line	6	21	36	47				

## Data Availability

The data presented in this study are available on request from the corresponding author.
